# Extracellular Vesicles as Natural Delivery Carriers Regulate Oxidative Stress Under Pathological Conditions

**DOI:** 10.3389/fbioe.2021.752019

**Published:** 2021-09-07

**Authors:** Hongzhao Qi, Yingruo Wang, Shunxin Fa, Changqing Yuan, Lijun Yang

**Affiliations:** ^1^ Department of Aging Research, Institute of Translational Medicine, The Affiliated Hospital of Qingdao University, College of Medicine, Qingdao University, Qingdao, China; ^2^ Shandong University of Science and Technology, Qingdao, China; ^3^ School of Stomatology, Qingdao University, Qingdao, China; ^4^ York School, Monterey, CA, United States; ^5^ Qingdao Institute of Bioenergy and Bioprocess Technology, Chinese Academy of Sciences, Qingdao, China

**Keywords:** extracellular vesicles, oxidative stress, delivery carriers, direct effects, indirect effects

## Abstract

Extracellular vesicles are cellular secretory particles that can be used as natural drug delivery carriers. They have successfully delivered drugs including chemotherapeutics, proteins, and genes to treat various diseases. Oxidative stress is an abnormal physiological phenomenon, and it is associated with nearly all diseases. In this short review, we summarize the regulation of EVs on oxidative stress. There are direct effects and indirect effects on the regulation of oxidative stress through EVs. On the one hand, they can deliver antioxidant substances or oxides to recipient cells, directly relieving or aggravating oxidative stress. On the other hand, regulate factors of oxidative stress-related signaling pathways can be delivered to recipient cells by the mediation of EVs, realizing the indirect regulation of oxidative stress. To the best of our knowledge, however, only endogenous drugs have been delivered by EVs to regulate oxidative stress till now. And the heterogeneity of EVs may complicate the regulation of oxidative stress. Therefore, this short review aims to draw more attention to the EVs-based regulation of oxidative stress, and we hope excellent EVs-based delivery carriers that can deliver exogenous drugs to regulate oxidative stress can be exploited.

## Introduction

Extracellular vesicles (EVs) are phospholipid bilayer-encapsulated vesicles that can be secreted by nearly all cell types (Andaloussi et al., 2013). They can be found in cell culture supernatants as well as biological fluids such as saliva (Winck et al., 2015), blood (Arraud et al., 2014), milk (Zonneveld et al., 2014), cerebrospinal fluids (Akers et al., 2016), and malignant ascites (Li et al., 2019). According to their biogenesis, EVs can be generally divided into three distinct categories: exosomes, microvesicles, and apoptotic bodies (Lässer et al., 2018). Exosomes originate from multivesicular bodies (MVBs) formed by the invagination of the limiting membrane of endosomes (Théry et al., 2002). MVBs can fuse with the cellular membrane to release exosomes into the extracellular space. Different from exosomes, microvesicles and apoptotic bodies are both generated from the cell membrane (Akers et al., 2013). Microvesicles derive from direct outward budding of the normal cells membrane, while apoptotic bodies are only secreted by dying cells during their fragmentation. Besides the origination, size is commonly used as a criterion to distinguish between the three types of EVs (Varga et al., 2014). In general, the size of exosomes, microvesicles, and apoptotic bodies is respectively 30–150 nm, 50–1,000 nm, and 50 nm-2 μm. It should be noted that the size range of the three types of EVs is overlapped due to their severe heterogeneity (Lőrincz et al., 2015). Furthermore, the heterogeneity of EVs may result in the absence of marker proteins belonging to a certain population. The distinguish of different types of EVs, therefore, cannot rely on a single standard, and it needs to comprehensively consider multiple criterions such as size, marker proteins or genes, and specific lipids (Théry et al., 2018). EVs have been recognized as a kind of messengers of intercellular communication during the past several decades. They can transport cargoes including DNA, RNA, proteins, and lipids between neighboring or distant cells (Tkach and Théry 2016). Taking advantage of their cargoes, EVs can perform numerous physiological functions. As an example, EVs can regulate immune responses to participate in the occurrence and development of many diseases such as cancers, cardiovascular diseases, osteoporosis, and central nervous system diseases (Robbins and Morelli, 2014; Yin et al., 2017).

**FIGURE 1:**
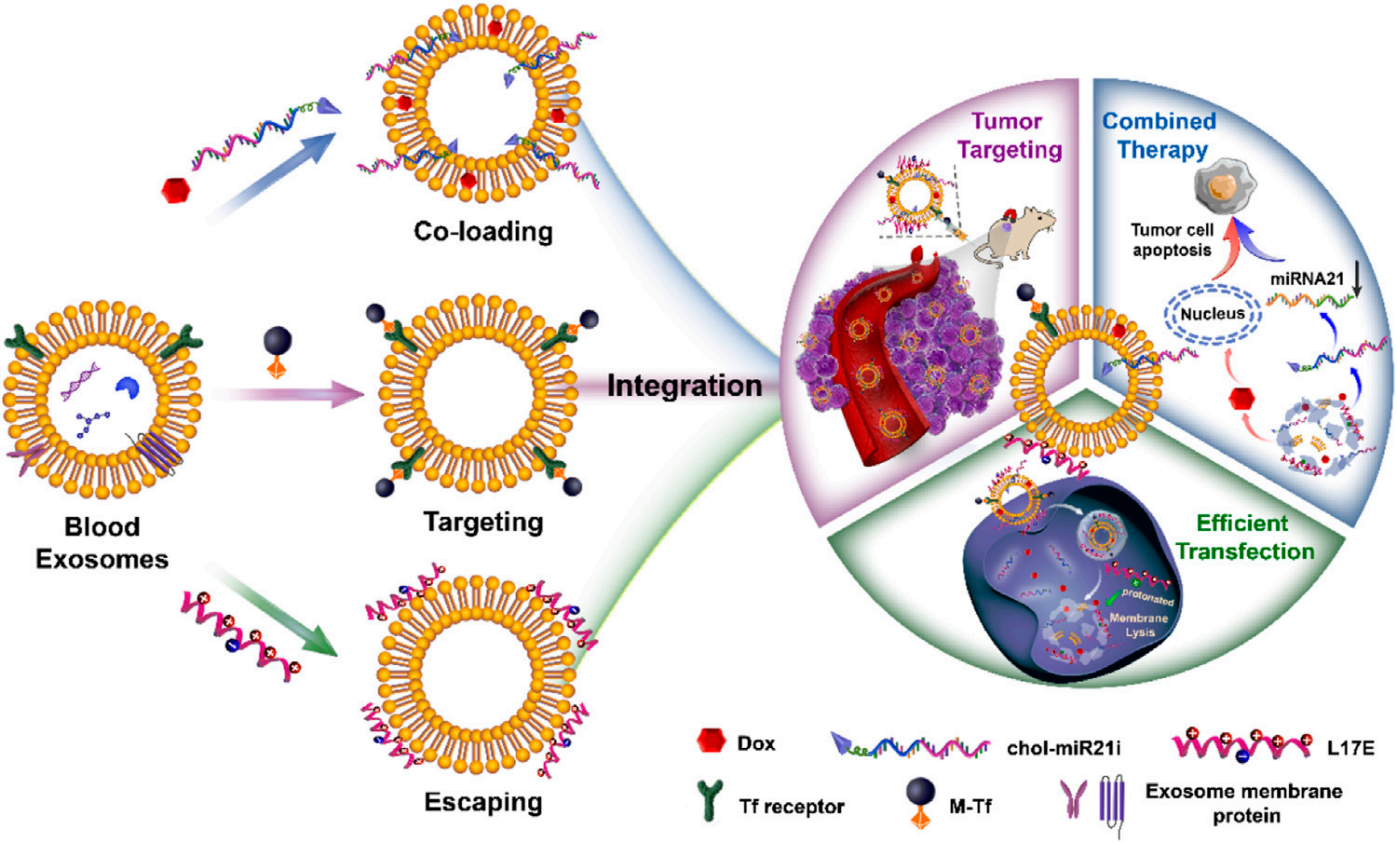
The preparation of engineered blood exosomes and their mechanism for the combined antitumor therapy with nucleic acid drugs and chemotherapeutics (Zhan et al., 2020).

From a material science point of view, EVs are natural drug delivery carriers. Their phospholipid bilayers similar in structure to liposomes endow EVs with the ability of hydrophilic and hydrophobic drugs loading (van der Meel et al., 2014). And the loaded drugs can be effectively protected from degradation. Compared with liposomes, EVs have intrinsic stability owing to their negatively charged surface and the support of skeletal proteins (Frank et al., 2018). Importantly, EVs have many unique properties superior to synthetic drug delivery carriers. They have low immunogenicity and cytotoxicity due to their endogenous source (Elsharkasy et al., 2020). And their lipid composition and protein content endow them with inherent active targeting properties and the capacity to cross multiple biological barriers such as the blood-brain barrier and mucosal barrier (Ashrafian et al., 2019; Matsumoto et al., 2017). Besides endogenous compositions, exogenous drugs can be loaded in EVs and delivered into target cells (Vader et al., 2016). In our previous researches, we have exploited a series of blood exosome-based delivery systems to efficiently deliver chemotherapeutics for tumor therapy (Qi et al., 2017; Qi et al., 2016; Qi et al., 2018; Yang et al., 2019; Zhan et al., 2020). As shown in Figure 1, we take advantage of the structure and properties of the blood exosome membrane to load nucleic acid drugs and chemotherapeutics. The system with the tumor-targeting ability and endosome-escaping capacity realizes the combined antitumor therapy. In general, EVs have been used as carriers to deliver endogenous or exogenous drugs for disease treatment. Oxidative stress is an abnormal physiological phenomenon, and it is defined as “an imbalance between antioxidants and oxidants, resulting in the disruption of redox signaling and the corresponding molecular damage.” To the best of our knowledge, it is associated with nearly all diseases such as cancers (Hayes et al., 2020), cardiovascular diseases (Dubois-Deruy et al., 2020), aortic dissection (Zong et al., 2021), atherosclerosis (Li et al., 2021; Xue et al., 2021), COVID-19 (Li et al., 2021), drug-induced injuries (Li et al., 2021), and diabetes (Zhang et al., 2020). The effective regulation or relief of oxidative stress can potentially prevent many diseases, and EVs play an important role in this process. In this mini-review, we will mainly summarize how oxidative stress under pathological conditions is modulated by EV-mediated drug delivery, and also discuss the influence of oxidative stress on EVs.

## EVs Modulate Oxidative Stress

**FIGURE 2:**
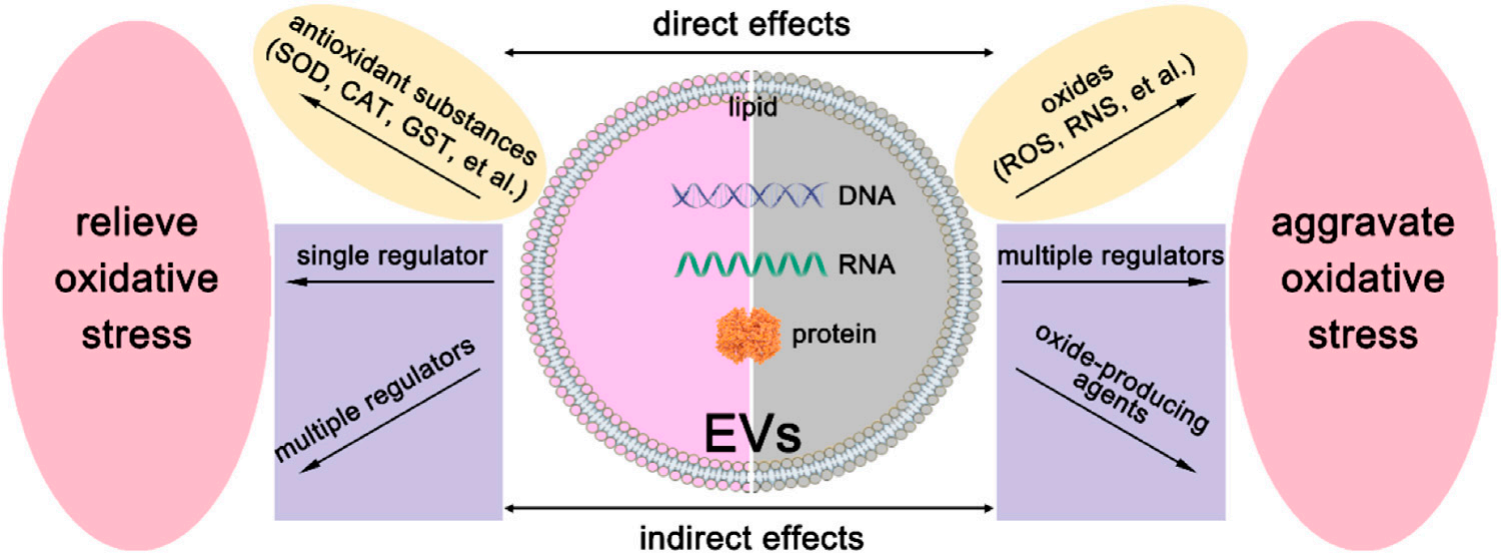
The modulation mechanism of oxidative stress by EVs. There are direct effects and indirect effects of EVs on the modulation of oxidative stress. To relieve oxidative stress, EVs can directly deliver antioxidant substances, such as SOD, CAT, GST, et al., and indirectly deliver a single regulation factor or multiple regulators to recipient cells. For the aggravation of oxidative stress, EVs can deliver oxides such as ROS and RNS to recipient cells directly. Besides, oxide-producing agents or multiple regulators can be delivered to recipient cells to realize indirect effects.

As we have mentioned above, EVs derived from different cells have heterogeneity. They may have different sizes, shapes, and compositions. Therefore, EVs may exhibit diverse regulations of oxidative stress, and the modulation mechanism is shown in Figure 2. On the one hand, they can deliver reactive oxygen species (ROS) scavengers to relieve oxidative stress. On the other hand, they may produce ROS by their detrimental contents. Below we will discuss these two aspects in great detail.

### EVs Relieve Oxidative Stress

Under normal physiological conditions, the production and elimination of ROS are balanced, while oxidative stress is produced when this balance is disturbed (Betteridge, 2000). In eukaryotic cells, antioxidant enzymes are commonly used as ROS scavengers to maintain the balance. EVs could inherently carry antioxidant enzymes, and numerous proteomic analyses have proved that superoxide dismutases (SOD), catalase (CAT), peroxiredoxin (PRDX), glutathione peroxidase (GPX), glutathione S-transferase (GST), and thioredoxin (TRX), et al. can be secreted into EVs (Bodega et al., 2019). These antioxidant enzymes loaded in EVs can directly relieve oxidative stress under various pathological conditions. For example, EVs released from T lymphocytes can deliver antioxidant enzymes, such as SOD isoforms and CAT, to human umbilical vein endothelial cells (HUVECs) and scavenge ROS (Soleti et al., 2012). Compared with other cells, stem cells are one of the main sources of antioxidant enzyme-loaded EVs. EVs derived from human mesenchymal stem cells (hMSCs) can protect hippocampal neurons from oxidative stress by the mediation of CAT (Bodart-Santos et al., 2019). hMSCs can also secret manganese-SOD (Mn-SOD) into EVs that can reduce oxidative stress in the hepatic ischemia-reperfusion injury (Yao et al., 2018). In addition to antioxidant enzymes, other proteins can also be riched in EVs to relieve oxidative stress. EVs secreted by astroglial cells can transport apolipoprotein D to neurons and reduce oxidative stress damage (Pascua-Maestro et al., 2019). Besides, endothelial EVs carry functional endothelial nitric oxide synthase (eNOS) to regulate eNOS/Akt signaling pathway, protecting against oxidative stress in endothelial cells (Mahmoud et al., 2017). These antioxidant substance-loaded EVs are more like ROS scavengers and they can directly eliminate both intracellular and extracellular ROS to relieve oxidative stress.

Besides the above direct effects, indirect effects on ROS or oxidative stress can be carried out by EVs. They can deliver drugs to target cells and regulate oxidative stress-associated signaling pathways. For example, EVs secreted by H293T cells can deliver microRNA-126 to H_2_O_2_-treated neonatal rat ventricular cardiomyocytes, attenuating the ROS elevation and reducing the apoptosis by targeting ERRFI1 (Wang et al., 2019). By comparison, stem cell-derived EVs possess a greater regulatory capacity owing to their versatility. EVs derived from hMSCs can mediate the nuclear factor erythroid 2-related factor-2 (Nrf2) signaling pathway to reduce ROS generation in H_2_O_2_-stimulated keratinocytes or UV-irradiated mice skin (Wang et al., 2020). They can also resist cisplatin-induced oxidative stress by increasing GSH levels and suppressing activation of the p38MAPK pathway (Zhou et al., 2013). Adipose-derived mesenchymal stem cells (ADMSCs)-derived EVs can alleviate LPS induced accumulation of ROS and inflammatory cytokines in macrophages *via* regulating the Nrf2/heme oxygenase-1 (HO-1) signaling pathway (Shen et al., 2021). Besides, they can reduce ischemia/reperfusion injury as well as lung injury by suppressing oxidative stress (Liu et al., 2019; Gao et al., 2021). EVs derived from human placental mesenchymal stem cells (hP-MSCs) can eliminate ROS and reduce the activity of myeloperoxidase (MPO) effectively suppressing oxidative stress (Duan et al., 2020). Human Wharton Jelly mesenchymal stem cells (hWJMSCs)-released EVs can protect the kidney against ischemia/reperfusion injury by suppressing nicotinamide adenine dinucleotide phosphate oxidases 2 (NOX2) expression or enhancing Nrf2/ARE activation and the subsequent alleviation of the oxidative stress (Zhang et al., 2014; Zhang et al., 2016a). These indirect regulation effects may be more lasting. That is because antioxidant substances loaded in EVs for the direct relief of oxidative stress are readily destroyed.

Compound types of EVs such as EVs separated from body fluids also show the capacity of oxidative stress relief. Human milk-derived EVs protect intestinal stem cells (ISCs) from oxidative stress injury mediated *via* the Wnt/β-catenin signaling pathway (Dong et al., 2020). Blood-derived EVs from healthy individuals can restore the homeostasis of oxidative stress in the mice model of Parkinson’s disease possessing neuroprotective effects (Sun et al., 2020). Camel milk EVs and their related genes ameliorate oxidative stress, normalize the antioxidant status, regulate the inflammatory pattern and improve the immune response in the cyclophosphamide (CTX)-treated animals (Ibrahim et al., 2019). The regulation mechanisms of compound types of EVs are more complicated because different types of EVs may have distinct functions. Furthermore, the ratio of different types of EVs is diverse at each separation resulting in the unstable regulation efficiency of compound types of EVs.

### EVs Aggravate Oxidative Stress

In addition to being therapeutics, EVs may also be accomplices of oxidative stress. They can directly load ROS or other oxides and deliver them to recipient cells. EVs derived from hypoxia/reoxygenation-treated HUVECs can deliver ROS into H9C2 cardiomyocytes resulting in ROS overload and subsequent oxidative stress (Zhang et al., 2016b). However, this direct effect mediated by EVs-loaded ROS is rarely reported owing to the extremely short life span of ROS. Similar to indirect relief of oxidative stress, the indirect aggravation of oxidative stress depends on the contents of EVs. On the one hand, EVs may directly produce ROS by the loaded enzymes to aggravate oxidative stress. As an example, endothelial EVs containing NOX subunits produce ROS to involve in endothelial damage (Burger et al., 2016). On the other hand, EVs can regulate target cells to indirectly aggravate oxidative stress. Gene drugs loaded in EVs have multi-target control ability, and they may induce numerous target genes to involve in oxidative stress. For example, EVs from ionizing irradiated mouse embryo fibroblasts (MEFs) can deliver microRNA-34c into unirradiated cells, potentially triggering a cascade of gene expression alterations to increase ROS and inducing bystander oxidative stress (Rastogi et al., 2018). And this radiation-induced bystander effect is also present in the body. EVs originated from mice irradiated with 2-Gy X-rays are intravenously injected into bystander mice, and the circulating reactive oxygen metabolite (ROM) level is increased (Hargitai et al., 2021). Ketamine-injured human uroepithelial cells (SV-HUC-1) cells can secrete EVs containing specific miRNAs. They enhance oxidative stress by mediating P38/NF-κB pathway (Xi et al., 2020). Besides genes, proteins or other small molecules can also be delivered by EVs. Polycyclic aromatic hydrocarbons-treated hepatocytes release EVs that are enriched in proteins, such as NOX and ferritin, as well as iron (van Meteren et al., 2020). These EVs could induce oxidative stress in recipient cells, thereby resulting in apoptosis.

It should be noted EVs that can aggravate oxidative stress are mainly derived from stimulated cells. The stimulating factors usually cause changes in the redox state of parent cells, and cells would secrete EVs to maintain their redox homeostasis. These EVs contain genes, proteins, and even lipids that can activate or regulate signaling pathways of oxidative stress aggravation. Therefore, these EVs can be thought of as products of the stress response of cells.

This stress response also occurs in the body. EVs derived from body fluids such as blood potentially aggravate oxidative stress. Platelet-derived EVs are the main type of EVs in the blood, and they are readily instigated to increase oxidative stress. In severe sepsis patients, platelet-derived EVs can produce ROS by the mediation of NOX and subsequently induce vascular cell apoptosis (Janiszewski et al., 2004). However, it is also reported that EVs derived from septic shock patients show protective effects on vascular function (Mostefai et al., 2008). This difference is potentially due to different sources of EVs. Besides, EVs from mice with the acetaminophen-induced liver injury can cause the allogeneic mice plasma ROS elevation inducing *in vivo* toxicity (Cho et al., 2018). Body fluids of different individuals are significant heterogeneity, and corresponding EVs may differ in origin, composition, and structure. Different from EVs from a specific type of cells, therefore, EVs separated from body fluids may show diverse functions.

## Oxidative Stress Modulate EVs

As drug delivery carriers, EVs can modulate oxidative stress by their contents. While the contents of EVs are readily changed under different pathological conditions. Cells with or without the treatment of oxidative stress secrete EVs with heterogeneity, and these diverse EVs may possess positive or negative effects on their adjacent or distant cells and tissues.

### Oxidative Stress Educates EVs

Under oxidative stress, cells may secrete EVs with positive effects. Firstly, oxidative stress-educated EVs may have the anti-oxidative ability. These EVs can relieve the cell’s oxidative stress as well as the oxidative stress of other cells or tissues. EVs derived from the non-pigmented ciliary epithelium (NPCE) which are exposed to oxidative stress can deliver protecting messages to activate major antioxidant genes and enhance SOD and CAT activity, protecting the trabecular meshwork (TM). Mouse mast cells exposed to oxidative stress release EVs, and these EVs transport mRNA to recipient cells providing them with resistance against oxidative stress (Tailleux et al., 2010). Besides relieving oxidative stress, other beneficial physiological effects can also be realized through oxidative stress-educated EVs. Retinal pigment epithelium (RPE) cells release higher amounts of EVs when they are exposed to oxidative stress. These EVs have a higher expression of vascular endothelial growth factor receptors (VEGFRs) in the surface membrane and have VEGFR mRNA in their internal cavity, promoting angiogenesis in endothelial cells (Atienzar-Aroca et al., 2016). Besides, oxidative stress-educated EVs can also involve in the muscle regeneration process. EVs released from oxidatively challenged myotubes have an increased loading of nucleic acid. They can decrease the diameter of the myotube, the mRNA levels of myogenin, and the expression of myosin heavy chain, leading to the proliferation of recipient myoblast (Guescini et al., 2017). EVs derived from stem cells often show beneficial effects, and oxidative stress may enhance these effects. The angiogenic and antimicrobial protein content of EVs derived from H_2_O_2_-treated ADMSCs was altered (Mayo et al., 2019). These EVs can enhance the viability of flaps and increase capillary density, improving tissue recovery. Taken together, these oxidative stress-educated EVs can be used as compensatory measures to minimize damage caused by oxidative stress.

### Oxidative Stress Miseducates EVs

Whether *in vitro* or *in vivo*, the cells can interact with each other, and EVs are important media of the interaction. Under pathological conditions, oxidative stress cause damage to cells, while these cells would expand the damage to other cells by their miseducated EVs. To date, immune cells are the main victims of oxidative stress-miseducated EVs, and immunosuppressive EVs are often released under oxidative stress. It has been reported that Jurkat and Raji cells release EVs containing NKG2D relative mRNA and proteins under thermal or oxidative stress. These EVs can reduce the cytotoxicity mediated by natural killer cell group 2D (NKG2D) receptor-ligand system and thus impair the functions of natural killer (NK) cells (Zimmer et al., 2011). Besides the specific type of cells, the body fluids of patients also contain immunosuppressive EVs. EVs separated from the blood of patients with rheumatoid arthritis contain oxidized phospholipids. These EVs can stimulate cells expressing Toll-like receptor 4 (TLR4), involving in inflammatory diseases (Manček-Keber et al., 2015). In addition, EVs have an important role in feto-maternal communication. Under oxidative stress, fetal cells-derived EVs can deliver unique cargos to parturition-associated uterine cells, inducing inflammation (Shahin et al., 2021). The effects of oxidative stress-miseducated EVs on the immune system need to be taken seriously since the immune system involves in nearly all pathological diseases. Furthermore, how to effectively avoid the effects is also worth study.

## Future and Prospect

Genes, proteins, and even lipids loaded in EVs can all be used as regulatory factors. In recent researches, however, EVs used to regulate oxidative stress are undecorated products, and it means that regulatory factors are endogenous components of EVs. These crude EVs have low regulation efficiency of oxidative stress. That is because EVs have heterogeneity, and parts of EVs may not have the regulation ability because of their lack of endogenous regulatory factors. Furthermore, it is complicated to adjust endogenous components of EVs for the regulation of oxidative stress. To improve the regulation efficiency, therefore, exogenous drugs possessing the efficient regulation ability of oxidative stress should be loaded into EVs. Numerous methods, such as electroporation and surfactant incubation, have been applied to load exogenous drugs into EVs. But it is still difficult to efficiently load macromolecular drugs into EVs, and there is a great need to exploit new drug loading methods. Moreover, it should be noted that EVs should not be oxidated during the drug loading process because oxidative stress potentially miseducates these EVs rendering opposite functions.

The sources of EVs applied for oxidative stress regulation should be carefully selected. As we have mentioned above, once parent cells are subjected to oxidative stress, EVs are potentially miseducated, and they may have side effects. To improve the safety of EVs, an appropriate source of EVs should be identified, and parent cells should not be under pathological conditions. *In vitro* cells are readily influenced by culture conditions, and corresponding EVs can be miseducated to a large extent. Therefore, the body fluids of healthy individuals are outstanding sources of EVs. As an example, EVs separated from the blood of healthy individuals show excellent biosafety and can be used for the delivery of chemotherapeutics. However, EVs separated from body fluids are often compound. Homogeneous types of EVs should be separated from the source, potentially minimizing the heterogeneity. Furthermore, we should make full use of the characteristics of EVs for different diseases. For example, specific types of EVs can target specific types of diseases, facilitating their loading of exogenous drugs to regulate oxidative stress.

In conclusion, EVs can be used as carriers to deliver endogenous or exogenous drugs for the modulation of oxidative, but the source and loading drugs of EVs should be charily selected. And how to reduce the impact of the heterogeneity of EVs should also be carefully considered.

## References

[B1] Akers J. C. Gonda D. Kim R. Carter B. S. Chen C. C. (2013). Biogenesis of Extracellular Vesicles (EV): Exosomes, Microvesicles, Retrovirus-Like Vesicles, and Apoptotic Bodies. J. Neurooncol. 113, 1–11. 10.1007/s11060-013-1084-8 23456661 PMC5533094

[B2] Akers J. C. Ramakrishnan V. Nolan J. P. Duggan E. Fu C.-C. Hochberg F. H. (2016). Comparative Analysis of Technologies for Quantifying Extracellular Vesicles (EVs) in Clinical Cerebrospinal Fluids (CSF). PLoS One 11, e0149866. 10.1371/journal.pone.0149866 26901428 PMC4763994

[B3] Andaloussi S. Mäger I. Breakefield X. O. Wood M. J. A. (2013). Extracellular Vesicles: Biology and Emerging Therapeutic Opportunities. Nat. Rev. Drug Discov. 12, 347–357. 10.1038/nrd3978 23584393

[B4] Arraud N. Linares R. Tan S. Gounou C. Pasquet J.-M. Mornet S. (2014). Extracellular Vesicles from Blood Plasma: Determination of Their Morphology, Size, Phenotype and Concentration. J. Thromb. Haemost. 12, 614–627. 10.1111/jth.12554 24618123

[B5] Ashrafian F. Shahriary A. Behrouzi A. Moradi H. R. Keshavarz Azizi Raftar S. Lari A. (2019). Akkermansia Muciniphila-Derived Extracellular Vesicles as a Mucosal Delivery Vector for Amelioration of Obesity in Mice. Front. Microbiol. 10, 2155. 10.3389/fmicb.2019.02155 31632356 PMC6779730

[B6] Atienzar-Aroca S. Flores-Bellver M. Serrano-Heras G. Martinez-Gil N. Barcia J. M. Aparicio S. (2016). Oxidative Stress in Retinal Pigment Epithelium Cells Increases Exosome Secretion and Promotes Angiogenesis in Endothelial Cells. J. Cel. Mol. Med. 20, 1457–1466. 10.1111/jcmm.12834 PMC4956947 26999719

[B7] Betteridge D. J. (2000). What Is Oxidative Stress? Metabolism 49, 3–8. 10.1016/S0026-0495(00)80077-3 10693912

[B8] Bodart-Santos V. de Carvalho L. R. P. de Godoy M. A. Batista A. F. Saraiva L. M. Lima L. G. (2019). Extracellular Vesicles Derived from Human Wharton's Jelly Mesenchymal Stem Cells Protect Hippocampal Neurons from Oxidative Stress and Synapse Damage Induced by Amyloid-β Oligomers. Stem Cel Res. Ther. 10, 1–13. 10.1186/s13287-019-1432-5 PMC6864996 31747944

[B9] Bodega G. Alique M. Puebla L. Carracedo J. Ramírez R. M. (2019). Microvesicles: ROS Scavengers and ROS Producers. J. Extracellular Vesicles 8, 1626654. 10.1080/20013078.2019.1626654 31258880 PMC6586107

[B10] Burger D. Turner M. Munkonda M. N. Touyz R. M. (2016). Endothelial Microparticle-Derived Reactive Oxygen Species: Role in Endothelial Signaling and Vascular Function. Oxidative Med. Cell Longevity 2016, 1–10. 10.1155/2016/5047954 PMC4893592 27313830

[B11] Cho Y.-E. Seo W. Kim D.-K. Moon P.-G. Kim S.-H. Lee B.-H. (2018). Exogenous Exosomes from Mice with Acetaminophen-Induced Liver Injury Promote Toxicity in the Recipient Hepatocytes and Mice. Sci. Rep. 8, 1–13. 10.1038/s41598-018-34309-7 30375433 PMC6207703

[B12] Dong P. Zhang Y. Yan D.-y. Wang Y. Xu X. Zhao Y.-c. (2020). Protective Effects of Human Milk-Derived Exosomes on Intestinal Stem Cells Damaged by Oxidative Stress. Cel Transpl. 29, 096368972091269. 10.1177/0963689720912690 PMC7444213 32193954

[B13] Duan L. Huang H. Zhao X. Zhou M. Chen S. Wang C. (2020). Extracellular Vesicles Derived from Human Placental Mesenchymal Stem Cells Alleviate Experimental Colitis in Mice by Inhibiting Inflammation and Oxidative Stress. Int. J. Mol. Med. 46, 1551–1561. 10.3892/ijmm.2020.4679 32945344 PMC7447323

[B14] Dubois-Deruy E. Peugnet V. Turkieh A. Pinet F. (2020). Oxidative Stress in Cardiovascular Diseases. Antioxidants 9, 864. 10.3390/antiox9090864 PMC7554855 32937950

[B45] Eldh M. Ekström K. Valadi H. Sjöstrand M. Olsson B. Jernås M. (2010). Exosomes Communicate Protective Messages during Oxidative Stress; Possible Role of Exosomal Shuttle RNA. PLoS One 5, e15353. 10.1371/journal.pone.0015353 21179422 PMC3003701

[B15] Elsharkasy O. M. Nordin J. Z. Hagey D. W. de Jong O. G. Schiffelers R. M. Andaloussi S. E. (2020). Extracellular Vesicles as Drug Delivery Systems: Why and How? Adv. Drug Deliv. Rev. 159, 332–343. 10.1016/j.addr.2020.04.004 32305351

[B16] Frank J. Richter M. de Rossi C. Lehr C.-M. Fuhrmann K. Fuhrmann G. (2018). Extracellular Vesicles Protect Glucuronidase Model Enzymes during Freeze-Drying. Sci. Rep. 8, 1–8. 10.1038/s41598-018-30786-y 30120298 PMC6098026

[B17] Gao Y. Huang X. Lin H. Zhao M. Liu W. Li W. (2021). Adipose Mesenchymal Stem Cell-Derived Antioxidative Extracellular Vesicles Exhibit Anti-Oxidative Stress and Immunomodulatory Effects under PM2.5 Exposure. Toxicology 447, 152627. 10.1016/j.tox.2020.152627 33161053

[B18] Guescini M. Maggio S. Ceccaroli P. Battistelli M. Annibalini G. Piccoli G. (2017). Extracellular Vesicles Released by Oxidatively Injured or Intact C2C12 Myotubes Promote Distinct Responses Converging toward Myogenesis. Int. J. Mol. Sci. 18, 2488. 10.3390/ijms18112488 PMC5713454 29165341

[B19] Hargitai R. Kis D. Persa E. Szatmári T. Sáfrány G. Lumniczky K. (2021). Oxidative Stress and Gene Expression Modifications Mediated by Extracellular Vesicles: An *In Vivo* Study of the Radiation-Induced Bystander Effect. Antioxidants 10, 156. 10.3390/antiox10020156 33494540 PMC7911176

[B20] Hayes J. D. Dinkova-Kostova A. T. Tew K. D. (2020). Oxidative Stress in Cancer. Cancer cell 38, 167–197. 10.1016/j.ccell.2020.06.001 32649885 PMC7439808

[B67] Hedlund M. Nagaeva O. Kargl D. Baranov V. Mincheva-Nilsson L. Mincheva-Nilsson L. (2011). Thermal- and Oxidative Stress Causes Enhanced Release of NKG2D Ligand-Bearing Immunosuppressive Exosomes in Leukemia/lymphoma T and B Cells. PLoS One 6, e16899. 10.1371/journal.pone.0016899 21364924 PMC3045385

[B21] Ibrahim H. M. Mohammed-Geba K. Tawfic A. A. El-Magd M. A. (2019). Camel Milk Exosomes Modulate Cyclophosphamide-Induced Oxidative Stress and Immuno-Toxicity in Rats. Food Funct. 10, 7523–7532. 10.1039/c9fo01914f 31674611

[B22] Janiszewski M. Do Carmo A. O. Pedro M. A. Silva E. Knobel E. Laurindo F. R. M. (2004). Platelet-derived Exosomes of Septic Individuals Possess Proapoptotic NAD(P)H Oxidase Activity: A Novel Vascular Redox Pathway*. Crit. Care Med. 32, 818–825. 10.1097/01.ccm.0000114829.17746.19 15090968

[B23] Lässer C. Jang S. C. Lötvall J. (2018). Subpopulations of Extracellular Vesicles and Their Therapeutic Potential. Mol. Aspects Med. 60, 1–14. 10.1016/j.mam.2018.02.002 29432782

[B24] Li Y. Zhou J. Zhuo Q. Zhang J. Xie J. Han S. (2019). Malignant Ascite-Derived Extracellular Vesicles Inhibit T Cell Activity by Upregulating Siglec-10 Expression. Cancer. Manag. Res. 11, 7123–7134. 10.2147/CMAR.S210568 31534365 PMC6681125

[B25] Li D. Yang Y. Wang S. He X. Liu M. Bai B. (2021). Role of Acetylation in Doxorubicin-Induced Cardiotoxicity. Redox Biol. 46, 102089. 10.1016/j.redox.2021.102089 34364220 PMC8350499

[B26] Li X. Yang Y. Wang Z. Jiang S. Meng Y. Song X. (2021). Targeting Non-coding RNAs in Unstable Atherosclerotic Plaques: Mechanism, Regulation, Possibilities, and Limitations. Int. J. Biol. Sci. 17, 3413–3427. 10.7150/ijbs.62506 34512156 PMC8416736

[B27] Li X. Yin D. Yang Y. Bi C. Wang Z. Ma G. (2021). Eosinophil: A Nonnegligible Predictor in COVID-19 Re-Positive Patients. Front. Immunol. 12, 690653. 10.3389/fimmu.2021.690653 34394084 PMC8358389

[B28] Liu Z. Xu Y. Wan Y. Gao J. Chu Y. Li J. (2019). Exosomes from Adipose-Derived Mesenchymal Stem Cells Prevent Cardiomyocyte Apoptosis Induced by Oxidative Stress. Cell Death Discov. 5, 1–7. 10.1038/s41420-019-0159-5 PMC6425027 30911413

[B29] Lorincz A. M. Schütte M. Timár C. I. Veres D. S. Kittel A. McLeish K. R. (2015). Functionally and Morphologically Distinct Populations of Extracellular Vesicles Produced by Human Neutrophilic Granulocytes. J. Leukoc. Biol. 98, 583–589. 10.1189/jlb.3VMA1014-514R 25986013

[B30] Mahmoud A. M. Wilkinson F. L. McCarthy E. M. Moreno‐Martinez D. M. Langford‐Smith A. Romero M. (2017). Endothelial Microparticles Prevent Lipid‐Induced Endothelial Damage via Akt/eNOS Signaling and Reduced Oxidative Stress. FASEB j. 31, 4636–4648. 10.1096/fj.201601244RR 28687612 PMC5714503

[B31] Manček-Keber M. Frank-Bertoncelj M. Hafner-Bratkovič I. Smole A. Zorko M. Pirher N. (2015). Toll-Like Receptor 4 Senses Oxidative Stress Mediated by the Oxidation of Phospholipids in Extracellular Vesicles. Sci. Signal. 8, ra60. 10.1126/scisignal.2005860 26082436

[B32] Matsumoto J. Stewart T. Banks W. A. Zhang J. (2018). The Transport Mechanism of Extracellular Vesicles at the Blood-Brain Barrier. Curr. Pharm. Des. 23, 6206–6214. 10.2174/1381612823666170913164738 28914201

[B33] Mayo J. S. Kurata W. E. O’Connor K. M. Pierce L. M. (2019). Oxidative Stress Alters Angiogenic and Antimicrobial Content of Extracellular Vesicles and Improves Flap Survival. Plast. Reconstr. Surg. - Glob. Open 7, e2588. 10.1097/gox.0000000000002588 32537316 PMC7288884

[B34] Mostefai H. A. Meziani F. Mastronardi M. L. Agouni A. Heymes C. Sargentini C. (2008). Circulating Microparticles from Patients with Septic Shock Exert Protective Role in Vascular Function. Am. J. Respir. Crit. Care Med. 178, 1148–1155. 10.1164/rccm.200712-1835OC 18723433

[B35] Pascua-Maestro R. González E. Lillo C. Ganfornina M. D. Falcón-Pérez J. M. Sanchez D. (2019). Extracellular Vesicles Secreted by Astroglial Cells Transport Apolipoprotein D to Neurons and Mediate Neuronal Survival upon Oxidative Stress. Front. Cel. Neurosci. 12, 526. 10.3389/fncel.2018.00526 PMC6335244 30687015

[B36] Qi H. Liu C. Long L. Ren Y. Zhang S. Chang X. (2016). Blood Exosomes Endowed with Magnetic and Targeting Properties for Cancer Therapy. ACS Nano 10, 3323–3333. 10.1021/acsnano.5b06939 26938862

[B37] Qi H. Jia H. Sang J. Ren Y. Zhao J. Hou X. (2017). Using Endogenous Ligands for Direct Superparamagnetic Nanoparticle Cluster-Based Body Fluid Exosome Separation. RSC Adv. 7, 2926–2933. 10.1039/C6RA24937J

[B38] Qi H. Yang L. Li X. Zhan Q. Han D. Zhao J. (2018). Exosomes Separated Based on the "STOP" Criteria for Tumor-Targeted Drug Delivery. J. Mater. Chem. B 6, 2758–2768. 10.1039/C8TB00355F 32254228

[B39] Rastogi S. Hwang A. Chan J. Wang J. Y. J. Luo K. (2018). Extracellular Vesicles Transfer Nuclear Abl-Dependent and Radiation-Induced miR-34c into Unirradiated Cells to Cause Bystander Effects. Mol. Biol. Cell. 29, 2228–2242. 10.1091/mbc.E18-02-0130 29975106 PMC6249796

[B40] Robbins P. D. Morelli A. E. (2014). Regulation of Immune Responses by Extracellular Vesicles. Nat. Rev. Immunol. 14, 195–208. 10.1038/nri3622 24566916 PMC4350779

[B41] Shahin H. I. Radnaa E. Tantengco O. A. G. Kechichian T. Kammala A. K. Sheller-Miller S. (2021). Microvesicles and Exosomes Released by Amnion Epithelial Cells under Oxidative Stress Cause Inflammatory Changes in Uterine Cells†. Biol. Reprod. 105, 464–480. 10.1093/biolre/ioab088 33962471 PMC8335356

[B42] Shen K. Jia Y. Wang X. Zhang J. Liu K. Wang J. (2021). Exosomes from Adipose-Derived Stem Cells Alleviate the Inflammation and Oxidative Stress *via* Regulating Nrf2/HO-1 axis in Macrophages. Free Radic. Biol. Med. 165, 54–66. 10.1016/j.freeradbiomed.2021.01.023 33476797

[B43] Soleti R. Lauret E. Andriantsitohaina R. Carmen Martínez M. (2012). Internalization and Induction of Antioxidant Messages by Microvesicles Contribute to the Antiapoptotic Effects on Human Endothelial Cells. Free Radic. Biol. Med. 53, 2159–2170. 10.1016/j.freeradbiomed.2012.09.021 23010499

[B44] Sun T. Ding Z.-X. Luo X. Liu Q.-S. Cheng Y. Hassanzadeh K. (2020). Blood Exosomes Have Neuroprotective Effects in a Mouse Model of Parkinson's Disease. Oxidative Med. Cell Longevity 2020, 1–14. 10.1155/2020/3807476 PMC7714585 33294121

[B46] Théry C. Zitvogel L. Amigorena S. (2002). Exosomes: Composition, Biogenesis and Function. Nat. Rev. Immunol. 2, 569–579. 10.1038/nri855 12154376

[B47] Théry C. Witwer K. W. Aikawa E. Alcaraz M. J. Anderson J. D. Andriantsitohaina R. (2018). Minimal Information for Studies of Extracellular Vesicles 2018 (MISEV2018): A Position Statement of the International Society for Extracellular Vesicles and Update of the MISEV2014 Guidelines. J. Extracell. Vesicles 7, 1535750. 10.1080/20013078.2018.1535750 30637094 PMC6322352

[B48] Tkach M. Théry C. (2016). Communication by Extracellular Vesicles: Where We Are and where We Need to Go. Cell 164, 1226–1232. 10.1016/j.cell.2016.01.043 26967288

[B49] Vader P. Mol E. A. Pasterkamp G. Schiffelers R. M. (2016). Extracellular Vesicles for Drug Delivery. Adv. Drug Deliv. Rev. 106, 148–156. 10.1016/j.addr.2016.02.006 26928656

[B50] van der Meel R. Fens M. H. A. M. Vader P. Van Solinge W. W. Eniola-Adefeso O. Schiffelers R. M. (2014). Extracellular Vesicles as Drug Delivery Systems: Lessons from the Liposome Field. J. Controlled Release 195, 72–85. 10.1016/j.jconrel.2014.07.049 25094032

[B51] van Meteren N. Lagadic-Gossmann D. Podechard N. Gobart D. Gallais I. Chevanne M. (2020). Extracellular Vesicles Released by Polycyclic Aromatic Hydrocarbons-Treated Hepatocytes Trigger Oxidative Stress in Recipient Hepatocytes by Delivering Iron. Free Radic. Biol. Med. 160, 246–262. 10.1016/j.freeradbiomed.2020.08.001 32791186

[B52] Varga Z. Yuana Y. Grootemaat A. E. Van der Pol E. Gollwitzer C. Krumrey M. (2014). Towards Traceable Size Determination of Extracellular Vesicles. J. Extracellular Vesicles 3, 23298. 10.3402/jev.v3.23298 PMC3916677 24511372

[B53] Wang W. Zheng Y. Wang M. Yan M. Jiang J. Li Z. (2019). Exosomes Derived miR-126 Attenuates Oxidative Stress and Apoptosis from Ischemia and Reperfusion Injury by Targeting ERRFI1. Gene 690, 75–80. 10.1016/j.gene.2018.12.044 30597234

[B54] Wang T. Jian Z. Baskys A. Yang J. Li J. Guo H. (2020). MSC-derived Exosomes Protect against Oxidative Stress-Induced Skin Injury via Adaptive Regulation of the NRF2 Defense System. Biomaterials 257, 120264. 10.1016/j.biomaterials.2020.120264 32791387

[B55] Winck F. V. Prado Ribeiro A. C. Ramos Domingues R. Ling L. Y. Riaño-Pachón D. M. Rivera C. (2015). Insights into Immune Responses in Oral Cancer through Proteomic Analysis of Saliva and Salivary Extracellular Vesicles. Sci. Rep. 5, 1–13. 10.1038/srep16305 PMC4633731 26538482

[B56] Xi X. J. Zeng J. J. Lu Y. Chen S. H. Jiang Z. W. He P. J. (2020). Extracellular Vesicles Enhance Oxidative Stress through P38/NF‐kB Pathway in Ketamine‐induced Ulcerative Cystitis. J. Cel. Mol. Med. 24, 7609–7624. 10.1111/jcmm.15397 PMC7339200 32441055

[B57] Xue Q. He N. Wang Z. Fu X. Aung L. H. H. Liu Y. (2021). Functional Roles and Mechanisms of Ginsenosides from Panax Ginseng in Atherosclerosis. J. Ginseng Res. 45, 22–31. 10.1016/j.jgr.2020.07.002 33437153 PMC7790891

[B58] Yang L. Han D. Zhan Q. Li X. Shan P. Hu Y. (2019). Blood TfR+ Exosomes Separated by a pH-Responsive Method Deliver Chemotherapeutics for Tumor Therapy. Theranostics 9, 7680–7696. 10.7150/thno.37220 31695794 PMC6831460

[B59] Yao J. Zheng J. Cai J. Zeng K. Zhou C. Zhang J. (2018). Extracellular Vesicles Derived from Human Umbilical Cord Mesenchymal Stem Cells Alleviate Rat Hepatic Ischemia‐Reperfusion Injury by Suppressing Oxidative Stress and Neutrophil Inflammatory Response. FASEB j. 33, 1695–1710. 10.1096/fj.201800131RR 30226809

[B60] Yin P. Lv H. Li Y. Deng Y. Zhang L. Tang P. (2017). Exosome-mediated Genetic Information Transfer, a Missing Piece of Osteoblast-Osteoclast Communication Puzzle. Front. Endocrinol. 8, 336. 10.3389/fendo.2017.00336 PMC5712011 29230197

[B61] Zhan Q. Yi K. Qi H. Li S. Li X. Wang Q. (2020). Engineering Blood Exosomes for Tumor-Targeting Efficient Gene/chemo Combination Therapy. Theranostics 10, 7889–7905. 10.7150/thno.45028 32685027 PMC7359100

[B62] Zhang G. Zou X. Miao S. Chen J. Du T. Zhong L. (2014). The Anti-Oxidative Role of Micro-Vesicles Derived from Human Wharton-Jelly Mesenchymal Stromal Cells through NOX2/gp91(phox) Suppression in Alleviating Renal Ischemia-Reperfusion Injury in Rats. PLoS One 9, e92129. 10.1371/journal.pone.0092129 24637475 PMC3956873

[B63] Zhang G. Zou X. Huang Y. Wang F. Miao S. Liu G. (2016a). Mesenchymal Stromal Cell-Derived Extracellular Vesicles Protect against Acute Kidney Injury through Anti-oxidation by Enhancing Nrf2/ARE Activation in Rats. Kidney Blood Press. Res. 41, 119–128. 10.1159/000443413 26894749

[B64] Zhang Q. Shang M. Zhang M. Wang Y. Chen Y. Wu Y. (2016b). Microvesicles Derived from Hypoxia/reoxygenation-Treated Human Umbilical Vein Endothelial Cells Promote Apoptosis and Oxidative Stress in H9c2 Cardiomyocytes. BMC Cel Biol. 17, 25. 10.1186/s12860-016-0100-1 PMC4919832 27338159

[B65] Zhang P. Li T. Wu X. Nice E. C. Huang C. Zhang Y. (2020). Oxidative Stress and Diabetes: Antioxidative Strategies. Front. Med. 14, 583–600. 10.1007/s11684-019-0729-1 32248333

[B66] Zhou Y. Xu H. Xu W. Wang B. Wu H. Tao Y. (2013). Exosomes Released by Human Umbilical Cord Mesenchymal Stem Cells Protect against Cisplatin-Induced Renal Oxidative Stress and Apoptosis *In Vivo* and *In Vitro* . Stem Cel Res. Ther. 4, 34. 10.1186/scrt194 PMC3707035 23618405

[B68] Zong T. Yang Y. Lin X. Jiang S. Zhao H. Liu M. (2021). 5′-tiRNA-Cys-GCA Regulates VSMC Proliferation and Phenotypic Transition by Targeting STAT4 in Aortic Dissection. Mol. Ther. - Nucleic Acids. in press 10.1016/j.omtn.2021.07.013 PMC8413832 34513311

[B69] Zonneveld M. I. Brisson A. R. van Herwijnen M. J. C. Tan S. van de Lest C. H. A. Redegeld F. A. (2014). Recovery of Extracellular Vesicles from Human Breast Milk Is Influenced by Sample Collection and Vesicle Isolation Procedures. J. Extracellular Vesicles 3, 24215. 10.3402/jev.v3.24215 PMC4139932 25206958

